# Effect of Silicone Modifier on the Physical Properties of Flexible Silica Aerogels

**DOI:** 10.3390/polym15092043

**Published:** 2023-04-25

**Authors:** Kun-Hao Luo, Min-Si Yan, Chen-An Chang, Chih-Wei Weng, Jui-Ming Yeh

**Affiliations:** Department of Chemistry, Chung Yuan Christian University, Chung Li District‚ Tao-Yuan City 32023, Taiwandan.chang8989@gmail.com (C.-A.C.);

**Keywords:** flexible, silica aerogel, modifier

## Abstract

Research on the development of flexible silica aerogels (FSAs) has been ongoing due to their excellent thermal insulation, low density, and high elasticity. However, the physical properties of FSAs, such as density, thermal conductivity, mechanical strength, and surface wettability, are highly dependent on the preparation conditions. To achieve the desired properties of FSAs for various applications, it is necessary to develop a method to fine-tune their physical properties. In this paper, two modifiers of methyltrimethoxysilane (MTMS)/trimethylethoxysilane (TMES) were employed to fine-tune the bulk density of a series of flexible silica aerogels (FSAs), reflecting a series of FSAs with fine-tunable physical properties. First, the precursor was synthesized by a click reaction between vinyltrimethoxysilane (VTMS) and 2,2′ (ethylenedioxy) diethanethiol (EDDET). The VTMS, EDDET, and the as-prepared precursor were characterized by FT-IR and NMR spectroscopy. Subsequently, the precursor was converted into a series of FSAs (denoted by FSA, FSA-M, and FSA-T) through conventional sol-gel reactions with/without MTMS/TMES. Chemical structures of synthesized FSAs were confirmed by ^13^C and ^29^Si solid-state NMR spectroscopy. The porous structure of FSAs was identified by BET and SEM, respectively. Physical properties, such as thermal conductivity, mechanical strength, and surface wettability of FSAs were determined by a Hot Disk, durometer/DMA in compression mode, and contact angle measurements, respectively. This study found FSAs containing none, 1 wt%, 5 wt%, and 10 wt% of MTMS increase the density of FSAs from 0.419 g/cm^3^ (FSA), 0.423 g/cm^3^ (FSA-M1), 0.448 g/cm^3^ (FSA-M5), and 0.456 g/cm^3^ (FSA-M10). It should be noted that the thermal conductivity, surface hardness, bulk mechanical strength, and hydrophobicity of FSA-Ms of increasing MTMS loading were all found to show a rising trend, while FSA-Ts exhibited lower density. FSA-T10 exhibited lower thermal conductivity, surface hardness, and bulk mechanical strength as compared to FSA. However, it was found to show higher hydrophobicity as compared to that of FSA.

## 1. Introduction

Silica aerogels of high transparency and flexibility are an emerging class of porous materials that plays a significant role in a spectrum of research areas [1,2,3,4,5]. This is a category of nano-porous material with many unique properties, such as low thermal conductivity, high porosity, hydrophobicity, high visible transparency, high specific surface area, and low dielectric constant [6,7,8,9,10,11]. The transparent nature of silica aerogels is contributed to by the size of the silica particle. It has a particle size that is smaller than the wavelength of visible light [12]. Moreover, to be classified as aerogel, it has to be of ultra-low density (porosity of >99%), making it a favorable material in thermal insulation [13], catalyst [14], chemical adsorption and absorption [15], chemical sensors [16], dielectric material [17], and medical applications (i.e., Drug delivery) [18].

Silica aerogel, prepared using a sol-gel reaction process, has been attracting considerable attention from scientists and engineers in different fields since it was first invented by S. S. Kistler in 1931 [19]. However, silica particles are weakly linked by nano-sized solid backbones, resulting in a brittle overall structure. Precursor wet gels need to be dried under supercritical conditions to minimize shrinkage and structural collapse [20,21], meaning the as-prepared aerogels are generally difficult to process or handled.

Extensive work has been done to develop a silica aerogel with high flexibility. Xu et al. [22] demonstrated the preparation of flexible bridged silsesquioxane aerogels produced from a thiol-ene click reaction of mercaptopropyltrimethoxysilane (MPTMS) and vinyltrimethoxysilane (VTMS). The soft thioether segments are obtained by a vacuum drying method with a reversible compression of up to 50% even after 20 repeats. Aravind et al. [23] Prepared flexible aerogels from methyltrimethoxysilane (MTMS) and 3-(2,3-epoxypropoxy)propyltrimethoxysilane (GPTMS) by employing a two-step acid–base sol–gel process along with the use of a surfactant. Resultant aerogels with a specific surface area of 0.104 g cm^−3^ were found with Young’s modulus to be as high as 0.46 MPa, as compared to Xu’s work, in which an aerogel of 0.085 g cm^−3^ in surface area was of 0.117 MPa.

The moisture sensitivity of aerogels is one other drawback from them being adopted in several applications. Replacing hydrophilic Si–OH groups with hydrolytically stable Si-R, where R = alkyl or aryl, groups inhibit the absorption of water. Therefore, MTMS containing one alkyl group is commonly chosen as a precursor to prepare hydrophobic silica aerogels. Theoretically, trimethylethoxysilane (TMES) consisting of three methyl groups should provide the overall aerogel with more hydrophobicity. While posing limiting the crosslinking points to the main chain, the flexibility of the aerogel is presumably reduced. Therefore, in this work, we attempt to prepare a series of flexible silica aerogels (FSAs) with tunable mechanical strength and varying hydrophobicity by using MTMS and TMES as silicone modifiers. First, the precursor was synthesized by a click reaction between vinyltrimethoxysilane (VTMS) and 2,2′-(ethylenedioxy)diethanethiol (EDDET). The VTMS, EDDET, and the as-prepared precursor were characterized by FT-IR and NMR spectroscopy. Subsequently, the precursor was converted into a series of FSAs (denoted by FSA, FSA-M, and FSA-T) through conventional sol-gel reactions with/without MTMS/TMES. Chemical structures of synthesized FSAs were confirmed by ^13^C and ^29^Si solid-state NMR spectroscopy. The porous structure of FSAs was identified by BET and SEM, respectively. Physical properties, such as thermal conductivity, thermal stability, mechanical strength, and surface wettability of FSAs, were determined by Hot Disk, TGA, durometer/DMA in compression mode, and contact angle measurements, respectively. The as-prepared silica aerogels open up a wide range of potential applications. Flexible silica aerogels with tunable mechanical strength can be used as insulation materials for pipes, walls, and roofs due to their low thermal conductivity [24]. The varying hydrophobicity of the aerogels can prevent moisture absorption, making them suitable for use in outdoor applications, such as coatings for buildings and vehicles [25].

## 2. Materials and Methods

### 2.1. Chemicals and Instrumentation

In this study, 2,2′-(ethylenedioxy)diethanethiol (EDDET, 95%, Aldrich, St. Louis, MO, USA), vinyltrimethoxysilane (VTMS, 98%, Aldrich), methyltrimethoxysilane (MTMS, 97%, Alfa Aesar, Haverhill, MA, USA), trimethylethoxysilane (TMES, 98%, Aldrich), n-hexane (98%, Riedel-deHaën, Seelze, Germany), hydrochloric acid (HCl, 37.0%, Riedel-deHaën), ammonia solution (NH_4_OH, 25%, Riedel-deHaën), and ethanol (>95%, J.T. Baker, Phillipsburg, NJ, USA) were used as received without further treatment.

Fourier Transform Infrared (JASCO FT, IR-4200, Oklahoma City, OK, USA) analysis was carried out at room temperature. ^1^H-NMR spectra run on a spectrometer (Bruker, Bruker 300, Billerica, MA, USA) using *d*-Chloroform (CDCl_3_) as a D-solvent, ^13^C-NMR, and ^29^Si-NMR spectra were acquired using a Solid-State NMR (Bruker, Bruker 400, Billerica, MA, USA) spectrometer. Brunauer Emmett-Teller (BET) data were obtained by performing nitrogen adsorption/desorption isotherms and accelerated surface area porosity analysis on Micromeritics ASAP-2010. The morphology of flexible silica aerogels was observed by Field-Emission Scanning electron microscopy (FE-SEM, JEOL, JSM-7600F, Akishima City, Japan). Contact angle measurements were carried out with Sindatek Instruments (First Ten Angstroms, FTA 125, Newark, NJ, USA). Mechanical properties were measured as follows: Dynamic Mechanical Analysis (DMA) was performed on a DuPont, TA Q800 (Delaware, DE, USA) to test the compression properties of materials, with loading gradually increasing from 0.01 N/min to 18 N/min. The hardness of the materials was measured by a Shore Hardness durometer (TECLOCK, GS-706N TYPE-A, Okaya-shi, Japan). A Hot Disk (HD, Hitachi, H5DR, Marunouchi, Japan) was used to measure the thermal conduction of the prepared samples, which was carried out using the measurement principle of the Transient Plane Source (TPS) Method by a Hot Disk Sensor (Design No. 5501) performed on a Keithley 2000 Muti-meter and Keithley 2400 Source-meter. Thermogravimetric analysis (TGA) was performed on a DuPont, TA Q50 (Delaware, DE, USA) by heating it from room temperature (~25 °C) to 800 °C at a heating rate of 5 °C × min^−1^.

### 2.2. Synthesis of Flexible Silica Aerogels

**Scheme 1:**
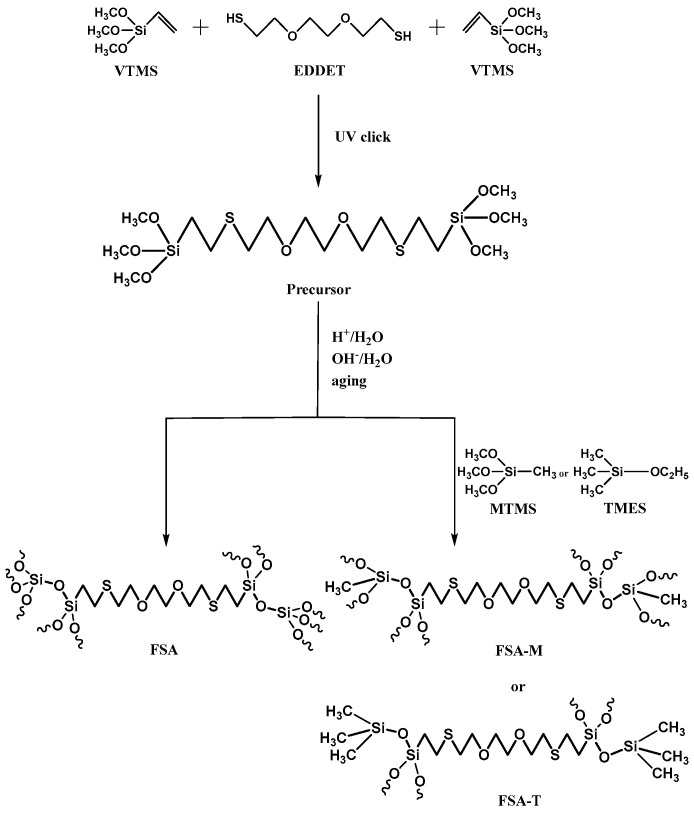
Flow chart for the preparation of flexible silica aerogels containing FSA, FSA-M, and FSA-T systems.

The representative flowchart to synthesize a standard flexible silica aerogel (denoted by FSA) and modified flexible silica aerogel (denoted by FSA-M and FSA-T) through sol-gel reaction is shown in Scheme 1. The typical procedure for the synthesis of FSA, FSA-M, and FSA-T is given as follows: First, the precursor was prepared by mixing 2 g (10.97 mmol) of EDT and 3.252 g (21.94 mmol) of VTMS, followed by magnetic stirring for 4 h under a UV light of 365 nm wavelength exposure at a distance of 10 cm. Afterward, 4.63 g of EtOH, 3.24 g of H_2_O, 0.4 g of 0.1 M HCl, and MTMS with different weight percentages (none, 1.0 wt%, 5.0 wt%, and 10.0 wt%), as well as TMES (10.0 wt%), were uniformly mixed. Subsequently, the mixed solution was added individually to the precursor to carry out the hydrolysis reaction. After 3 h of gentle stirring, a mixture of 3.24 g of H_2_O and 0.5 g of 0.1 M NH_4_OH was slowly added into the hydrolyzed solution in a dropwise manner and the solution was further stirred until even. After resting for 72 h, the solution was fully converted into a gel, followed by aging for another 48 h to produce a block of white gel. This was followed by immersing the gel in EtOH for 2 days. Afterward, n-hexane was used as a solvent exchanger to further immerse the gel for an additional 2 days before removing the solvent under heat treatment. The programmed heating conditions were as follows: 50 °C, 1 h; 70 °C, 1 h; and 80 °C, 1 h. The corresponding products of flexible silica aerogels were obtained and denoted as FSA, FSA-M1, FSA-M5, FSA-M10, and FSA-T10.

## 3. Results and Discussion

### 3.1. Material Characterization

#### 3.1.1. Spectroscopic Studies (FTIR and NMR) of EDDET, VTMS, Precursor

**Figure 1:**
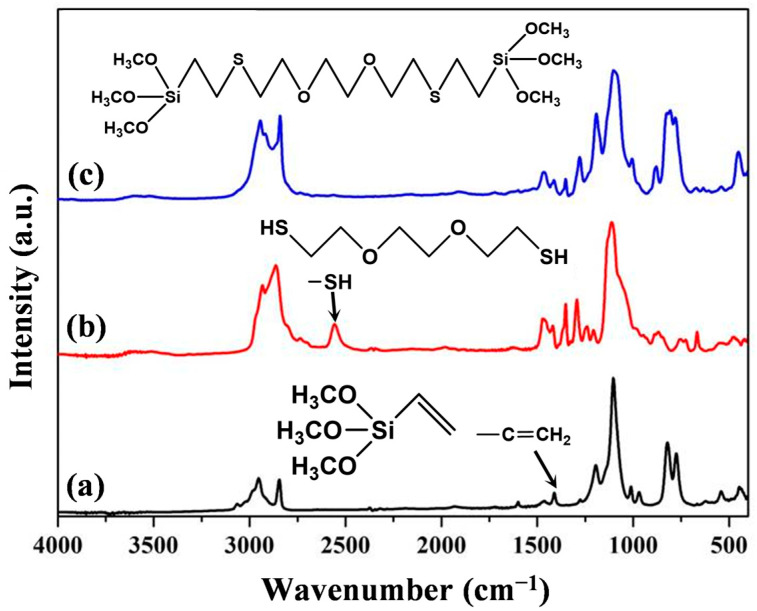
FTIR spectroscopy of (**a**) VTMS, (**b**) EDDET, and (**c**) the precursor of flexible silica aerogel.

**Figure 2:**
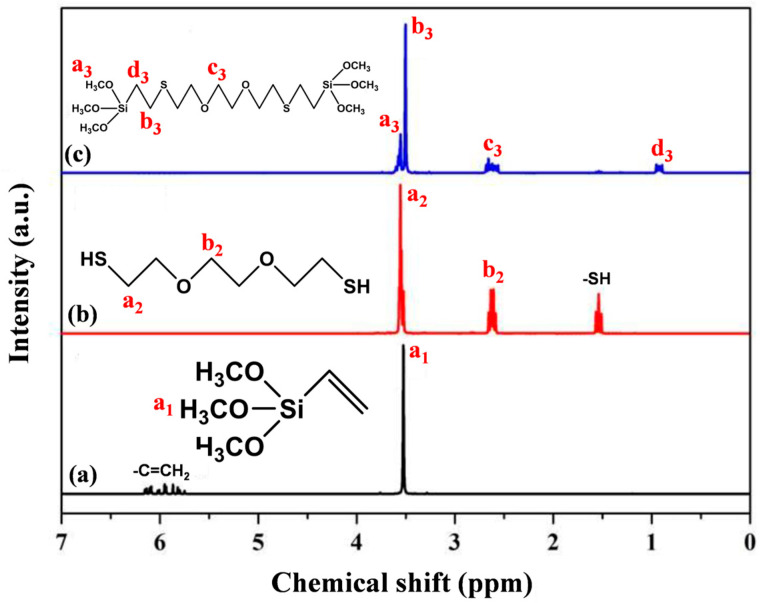
^1^H NMR spectra of (**a**) VTMS, (**b**) EDDET, and (**c**) the precursor of flexible silica aerogel where a_1_ was assigned to H signal of -CH_3_ in VTMS; a_2_ and b_2_ were assigned to H signals of -CH_3_ and -OCH_2_ in EDDET; a_3_, b_3_, c_3_ and d_3_ were assigned to H signals of -CH_3_, -SCH_2,_ -OCH_2_ and -SiCH_2_ in the precursor of flexible silica aerogel.

The representative FTIR spectra of EDDET, VTMS, and the as-prepared precursor are shown in Figure 1. In Figure 1a, the characteristic peak found at the position of 1075 cm^−1^ corresponded to the C=C stretching, and the sharp signal at 2840 cm^−1^ is due to asymmetric stretching in the Si-OCH_3_ of VTMS [26]. Moreover, the characteristic bands located at the position of 2556 cm^−1^, may be ascribed to the S-H stretching band of EDDET, as exhibited in Figure 1b. After the photochemical reaction, the representative FTIR spectra of the as-prepared precursor were generated by the addition and multiplication reaction between the reactants, as shown in Figure 1c. It should be noted that the vinyl peak of VTMS and the thiol signal of EDDET disappeared after 4 h under UV irradiation. It confirmed the formation of the target precursor. The representative ^1^H-NMR spectra of reactants (VTMS & EDDET) and the as-prepared precursor were shown in Figure 2. In Figure 2a, the VTMS was found to exhibit δ = 6.11 and δ = 3.54 ppm, which may be assigned to be the characteristic signal of the vinyl group (-CH=CH_2_) and the methoxy group (-OCH_3_), respectively. Moreover, in Figure 2b, EDDET was found to show the characteristic signal at the position of δ = 1.34 ppm, which may be attributed to the characteristic signal of the thiol group (-SH) [27]. The representative 1H-NMR spectrum of the as-prepared precursor was found with disappearances of δ = 6.11 and δ = 1.34 ppm, as shown in Figure 2c. This further confirmed the formation of the precursor.

#### 3.1.2. Characterization of Flexible Silica Aerogels (FSA, FSA-M, and FSA-T)

##### Spectroscopic Studies of Flexible Silica Aerogels (FSAs)

**Figure 3:**
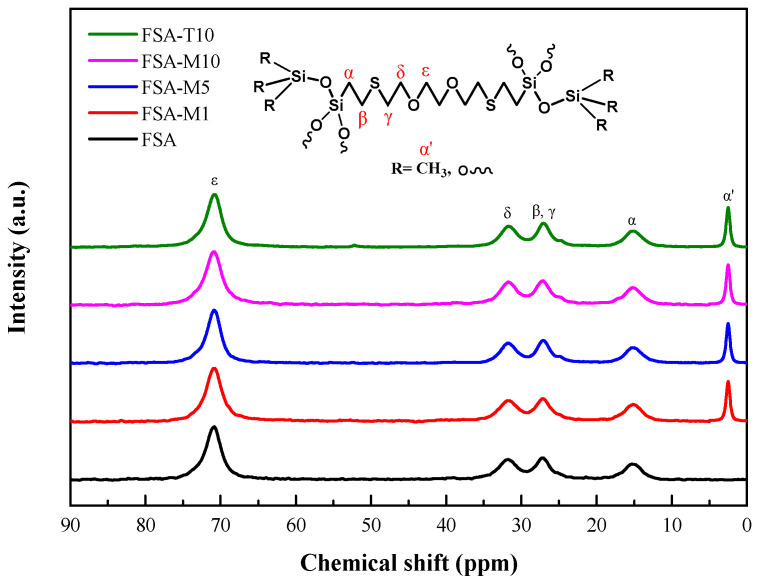
^13^C NMR spectra of FSA, FSA-M, and FSA-T.

To characterize the chemical structure of as-prepared flexible silica aerogels through a conventional sol-gel process with/without modification, solid-state ^13^C and ^29^Si-NMR spectra were used. Figure 3 shows the representative spectra of the ^13^C NMR spectra of as-prepared flexible silica aerogels (i.e., FSA, FSA-M1, FSA-M5, FSA-M10, and FSA-T10). It should be noted that all spectra exhibited characteristic signals of -CH_2_- (denoted by α, β, γ, δ, ε) values at the positions of δ = 14.6, 27.6, 32.4, and 71.4 ppm, and in FSA-M and FSA-T, additionally showed signals of -SiCH_3_ (denoted by α′) values at the position of δ = 2.5 ppm [27].

**Figure 4:**
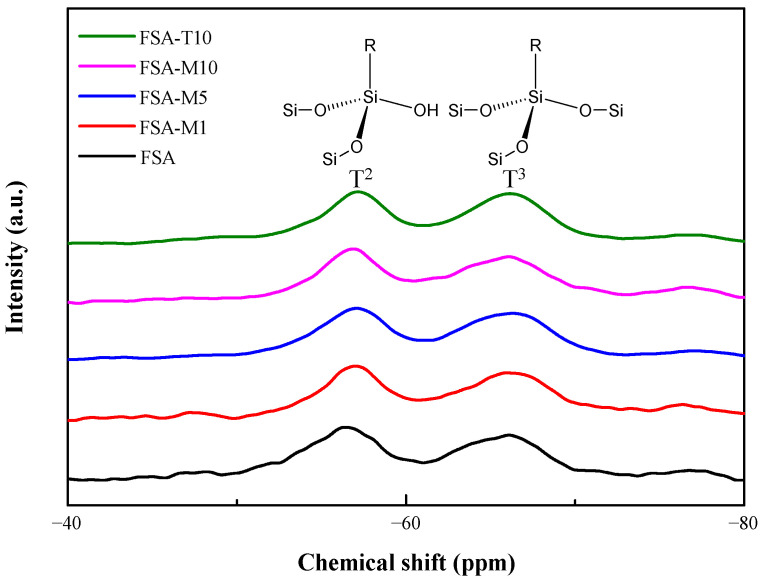
^29^Si NMR spectra of FSA, FSA-M and FSA-T.

Moreover, Figure 4 showed the representative spectra of the ^29^Si-NMR spectra of as-prepared flexible silica aerogels (i.e., FSA, FSA-M1, FSA-M5, FSA-M10, and FSA-T10). For example, the characteristic signal values of -Si(OSi)_3_ (denoted by T3) and -Si(OSi)_2_OH (denoted by T^2^) can be assigned at the positions of −67.1 ppm and −57.5 ppm, respectively [28].

##### Porous Structure Characterization of Flexible Silica Aerogels

**Figure 5:**
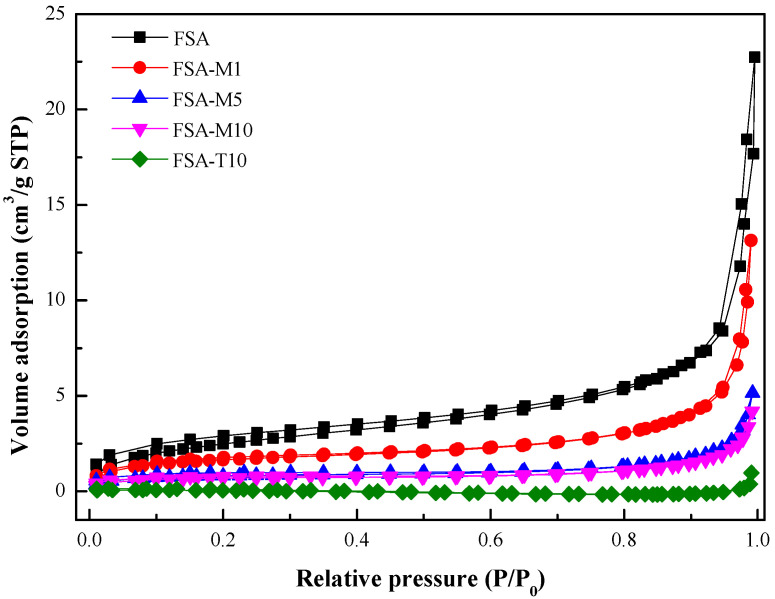
N_2_ adsorption/desorption isotherms of FSA, FSA-M1, FSA-M5, FSA-M10, and FSA-T10.

The surface area and pore volume of as-prepared flexible silica aerogels was determined by the N_2_ adsorption–desorption isotherm measurements of Brunauer–Emmett–Teller (BET) [29]. In this study, the N_2_ adsorption–desorption isotherms of as-prepared flexible silica aerogels with different MTMS and TMES contents were found to be Type IV-Langmuir Type [30], as shown in Figure 5, and the related data was summarized in Table 1. For example, the specific surface area and average pore diameter of FSA were found to be 9.41 m^2^/g and 30.7 nm, respectively. After FSA was modified with 1 wt% of MTMS, the specific surface area and average pore diameter of the FSA-M1 were decreased significantly down to 3.21 m^2^/g and 20.4 nm, respectively. It is reasonable to speculate that the higher surface area and pore size of FSA were attributed to the existence of a long linear chain moiety of a precursor. Upon the addition of MTMS, the sol-gel reaction occurred as the trimethoxysilane of the precursor reacted with the trimethoxysilane of MTMS, which may lead to the formation of a final flexible silica aerogel (i.e., FSA-M1) with higher (crosslinking) density as compared to that of FSA. The bulk density increases with the increase of crosslink density for crosslinked polymers [31]. FSA-M1 containing the silica resulted from the sol-gel reactions of MTMS, which may lower the overall ratio of the precursor, leading to the decrease of the specific surface area and pore diameter of flexible silica aerogels. The more loading of MTMS, the smaller the specific surface area and pore diameter of FSAs. For example, both the specific surface area and average pore diameter of the FSA-M10 decreased significantly down to 1.18 m^2^/g and 13.3 nm, respectively, as shown in Table 1. On the other hand, FSA modified with 10 wt% of TMES (FSA-T10) was found to have the specific surface area further decrease to 0.23 m^2^/g. However, the pore diameter of FSA-T10 was found to be 26.7 nm. This may be explained as follows: Upon the addition of TMES, the sol-gel reactions between the trimethoxysilane of the precursor molecules may be lowered or end-capped by the TMES molecules, which may effectively lower the crosslinking density of as-prepared flexible silica aerogels, hence being found with reduced bulk density.

### 3.2. Surface Morphology

**Figure 6:**
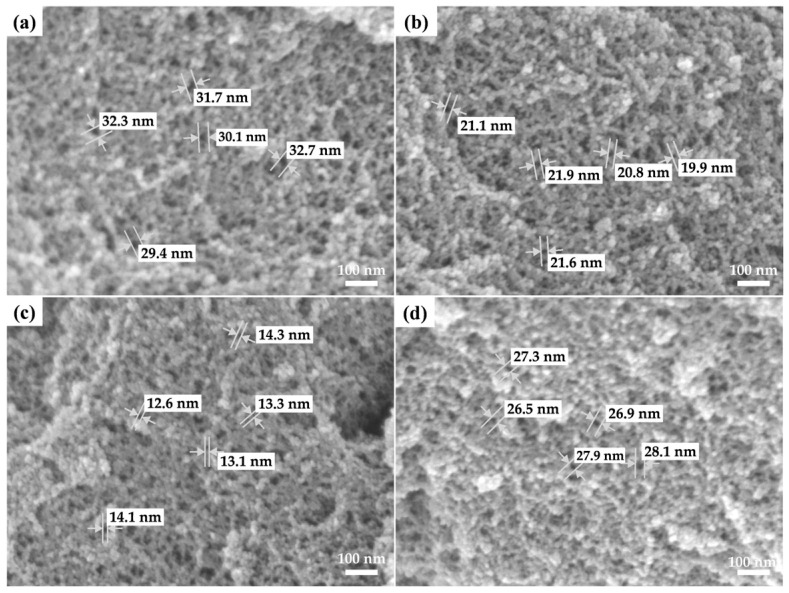
SEM images with magnifications of ×100,000 of (**a**) FSA, (**b**) FSA-M1, (**c**) FSA-M10, and (**d**) FSA-T10.

In this study, the surface morphology of FSAs was observed by SEM at a magnification of ×100,000, as shown in Figure 6. In Figure 6a, the surface morphology of FSA (without incorporating a silicone modifier) was found to exhibit an obvious porous structure with an average pore diameter of ~30 nm (Table 1). Upon the addition of 1 wt% and 10 wt% of MTMS, the surface morphology of FSA-M1 and FSA-M10 (Figure 6b,c) were found to show porous structures with smaller average pore diameters of ~20 nm and ~13 nm (Table 1), respectively. At the same time, an aerogel synthesized with the addition of 10 wt% of TMES, FSA-T10 (Figure 6d) was found to exhibit porous structures with an average pore diameter of ~27 nm (Table 1). Surface morphology images of the as-prepared flexible aerogels were consistent with the corresponding bulk density, as the collective data listed in Table 2 displays.

### 3.3. Thermal Properties of FSAs

#### 3.3.1. Thermal Conductivity (k) of FSAs

**Figure 7:**
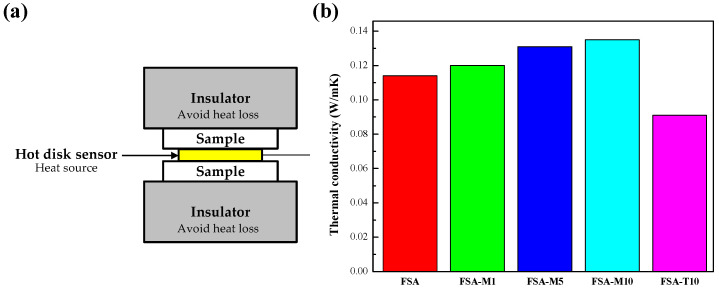
(**a**) Schematic diagram of thermal conductivity measurement, (**b**) thermal conductivity bar chart of FSA (red), FSA-M1 (green), FSA-M5 (blue), FSA-M10 (cyan), and FSA-T10 (magenta).

In this study, the thermal conductivity (k) of as-prepared flexible silica aerogels was determined by the Hot Disk technique, as illustrated in Figure 7a. The test was done with two test sample pieces cut of the same thickness and dimension, in this case, 10 mm in height and 16.8 mm in diameter, with an insulating cover at both ends to avoid heat loss. A controllable hot disk sensor is placed in between the two sample pieces, providing a heat source at the same time as taking a measurement of the k value, based on the Transient plane source method, TPS [32]. The bar chart for the k values of flexible aerogels was shown in Figure 7b and summarized in Table 2. First, the k value of FSA was 0.114 W/m°K. Upon the addition of 1 wt% of MTMS, the k value of FSA-M1 increased up to 0.120 W/m°K. This may be attributed to the lower specific surface area of FSA-M1 as compared to that of FSA, implying that a reduced amount of air (k of air = ~0.0257 W/m°K [32]) is trapped in FSA-M1, which leads to a higher k value. By increasing the loading of MTMS, the k value of FSA-M5 and FSA-M10 was further elevated to 0.131 and 0.135 W/m°K, respectively. On the other hand, the k value of FSA-T10 decreased down to 0.091 W/m°K. This reduced k value of FSA-T10 may be attributed to the end-capped sol-gel reaction of the precursor with the addition of TMES. Therefore, the majority of the trimethoxysilyl groups of the precursor did not mediate the sol-gel reaction, which leads to a lower specific surface area, which in turn results in higher air content within the aerogel and thus a better-insulated material with low thermal conductivity.

#### 3.3.2. Thermal Stability of FSAs

**Figure 8:**
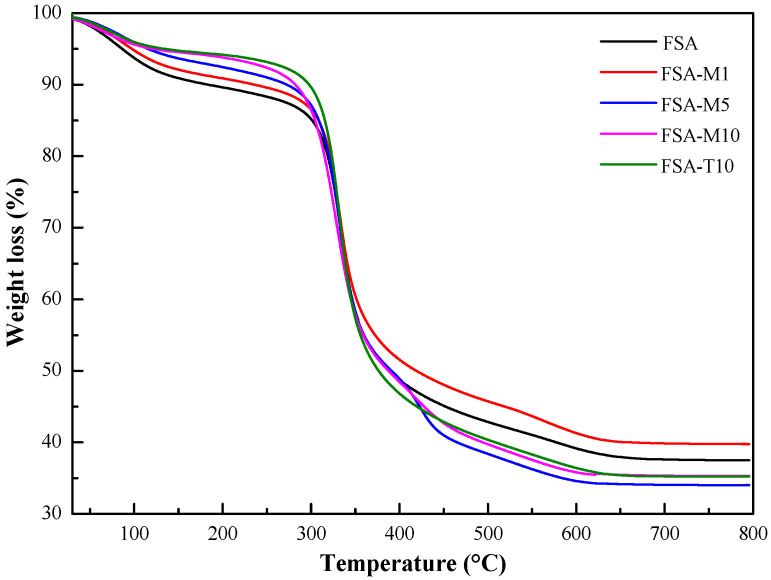
Thermogravimetry of FSA, FSA-M1, FSA-M5, FSA-M10, and FSA-T10.

The thermal stability of flexible silica aerogel and its modified counterparts, FSA-M1, FSA-M5, FSA-M10, and FSA-T10, were evaluated using thermogravimetric analysis (TGA) under atmospheric gas. Samples were heat-treated from room temperature (~25 °C) to 800 °C at a heating rate of 5 °C min^−1^, and the weight loss (%) as a function of the temperature was recorded (Figure 8). The TGA curve of all FSAs showed an obvious thermal decomposition stage between 100–300 °C, which was attributed to the thermal decomposition of the hydrophobic groups (–CH_3_ groups) [33]. It was noted that the hydrophobic silica aerogels displayed a higher decomposition temperature at 5 wt% loss (T_5d_), with FSA-T10 exhibiting the highest T_5d_ (135.33 °C), followed by FSA-M10 (121.77 °C), FSA-M5 (115.98 °C), FSA-M1 (97.45 °C), and FSA (86.46 °C). The results indicate that the presence of more –CH_3_ groups can lead to improved thermal stability. These findings suggest that modifying the surface of silica aerogels with hydrophobic groups could be a promising approach toward enhancing their thermal stability.

### 3.4. Mechanical Properties of FSAs

Mechanical properties study as-prepared FSAs and can classify them by surface hardness and bulk mechanical strength, which can be determined by a durometer and dynamic mechanical analysis (DMA) in compression mode, respectively.

#### 3.4.1. Surface Hardness of FSAs Determined by Durometer

**Figure 9:**
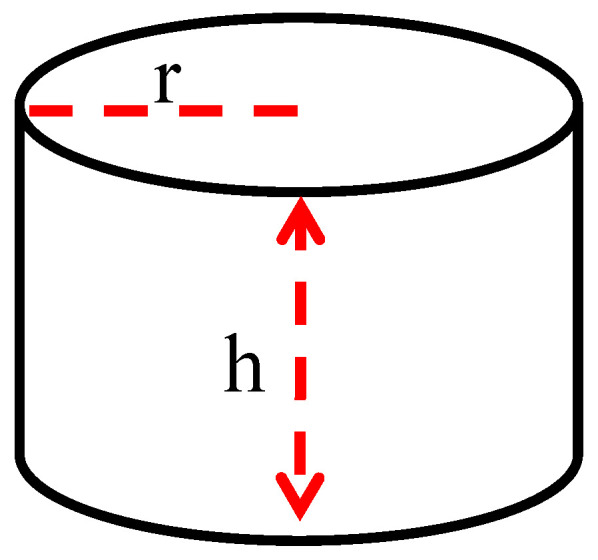
Schematic diagram of FSAs sample measurement.

In this section, the Equation (1) below was used to calculate the bulk density of the sample to make a preliminary judgment on the structural strength and structural cross-linking degree of the sample (Figure 9):

(1) $$D=\frac{M\left(g\right)}{V\left({\mathrm{cm}}^{3}\right)},V=r^{2}h\pi$$

where: *D* is the bulk density, *M* is the mass measured, *V* is the calculated volume of the prepared sample, *r* is the radius, and *h* is the height.

After indenting the sample with the pointer from the durometer, the hardness of the sample can be measured and obtained from the hardness tester. The bulk density and surface hardness of the samples are summarized in Table 2. The density of FSA was 0.419 g/cm^3^. Upon the addition of 1 wt% of MTMS, the density of FSA-M1 increased up to 0.423 g/cm^3^. By increasing the loading of MTMS, the density of FSA-M5 and FSA-M10 increased up to 0.448 and 0.456 g/cm^3^, respectively. However, the density of FSA-T10 decreased down to 0.333 g/cm^3^. As can be seen from the results in Table 2, the surface hardness of flexible aerogels has shown a similar trend to that of density. The hardness trend of as-prepared flexible silica aerogels: FSA-M10 (67 N/mm^2^) > FSA-M5 (55 N/mm^2^) > FSA-M1 (35 N/mm^2^) > FSA (34 N/mm^2^) > FSA-T10(20 N/mm^2^).

#### 3.4.2. Bulk Mechanical Strength of FSAs Determined by DMA in Compression Mode

**Figure 10:**
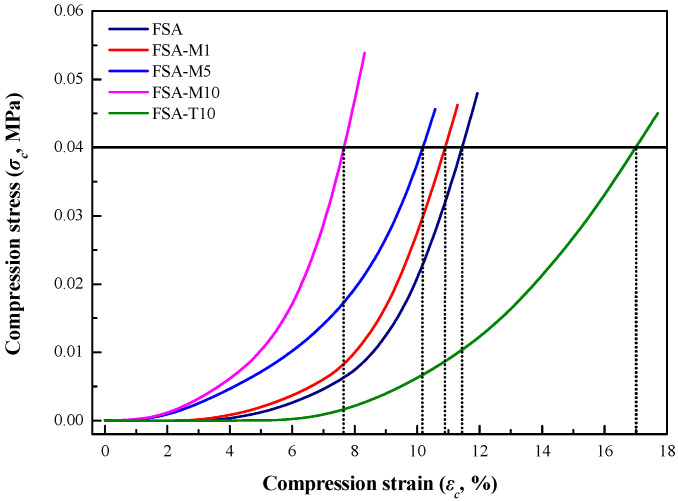
DMA stress-strain curves for FSA, FSA-M1, FSA-M5, FSA-M10, and FSA-T10.

The bulk mechanical properties of flexible silica aerogels were measured by DMA in compression mode (Figure 10). Samples with the shape of a cylinder, a height of ~10 mm, and a diameter of ~15 mm were used for testing. The stress-strain curve was plotted by DMA in compression mode with a fixed compression stress of 0.04 MPa. The results are summarized in Table 2. The larger the value of the compression strain percentage of flexible aerogels, the lower the mechanical strength of the corresponding flexible aerogels, which may have resulted from the lower (crosslinking) density of the as-prepared sample. As shown in Figure 8, under the fixed compression, the compression strain percentage of flexible silica aerogels was found to exhibit the following trend: FSA-T10 (16.99%) > FSA (11.44%) > FSA-M1 (10.89%) > TGA-M5 (10.18%) > FSA-M10 (7.65%).

### 3.5. Surface Wettability of FSAs Determined by CA Measurement of Water Droplets

**Figure 11:**
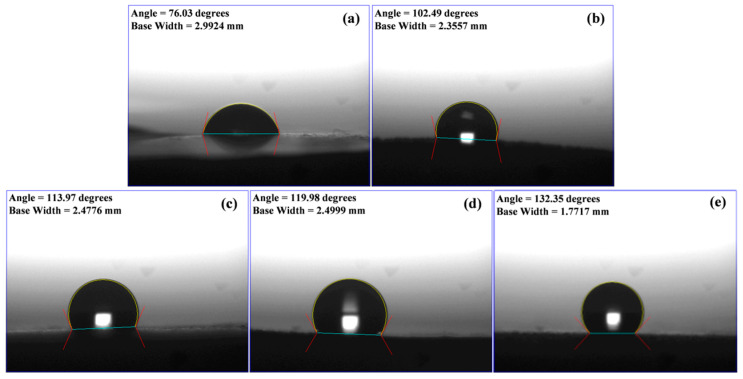
Contact angle of water droplets on (**a**) FSA, (**b**) FSA-M1, (**c**) FSA-M5, (**d**) FSA-M10, and (**e**) FSA-T10.

The surface wettability of flexible silica aerogels with and without silicone modifiers was determined by the contact angle (CA) measurements of water droplets, as shown in Figure 11 and summarized in Table 2. First, the CA of FSA was found to be ~76°. Upon the addition of the different loading of MTMS, the CA of FSA-M1, FSA-M5, and FSA-M10 were found to be ~102°, ~114°, and ~120°, respectively. The increasing CAs of water droplets upon the surface of flexible silica aerogels may be attributed to the following two reasons. First, a higher density of corresponding flexible silica aerogels may lead to a higher CA value of the water droplets. Second, the chemical structure of incorporated MTMS molecules possesses one hydrophobic methyl group. On the other hand, upon the addition of TMES, the CA of FGA-T10 was found to be ~132°. The significant increase in the CA of water droplets, from 76° of FGA to 132° of FGA-T10, may be attributed to the chemical structure of incorporated TMES molecules possessing three hydrophobic methyl groups [34].

### 3.6. Comparison & Application Prospect of As-Prepared Modified FSAs

The unique features of the hydrophobic silica aerogel produced in this study are listed in Table 3 alongside recent literature reports which have addressed the preparation of similar silica aerogels. Despite slightly higher thermal conductivity and density, the aerogel exhibited lowers Young’s modulus, emphasizing its enhanced elasticity [35]. This aspect is particularly beneficial, as materials which lower Young’s modulus have higher deformation capabilities, making them ideal for applications where flexibility and adaptability are required. Additionally, the slightly higher thermal conductivity and density levels are negligible compared to the significant advantages offered by the tunable mechanical strength and adjustable hydrophobicity of the modified silica aerogel. Overall, the results suggest that the modified silica aerogel developed in this study has the potential to outperform other aerogels in a range of applications, particularly those requiring enhanced elasticity, hydrophobicity, and mechanical robustness. Hydrophobic silica aerogels with enhanced elasticity and mechanical robustness are ideal for applications that require flexibility and adaptability. For example, it can be used as a flexible and durable coating for electronic devices to protect them from external stress and loading, isolating them from humidity effects [36]. In the automotive industry, they can be used as lightweight and durable components in cars, such as shock absorbers and bumpers, which need to withstand impact and vibration [37]. Additionally, in the sports industry, hydrophobic silica aerogels with tunable mechanical strength can be used to make protective gear, such as helmets, pads, and gloves, that offer both comfort and safety to athletes [38]. The biomedical industry can also benefit from these aerogels, particularly in tissue engineering applications where they can provide a scaffold for cell growth and regeneration [39]. The potential applications of hydrophobic silica aerogels with enhanced properties are vast, and their continued development could lead to the creation of high-performance materials that are versatile, lightweight, and adaptable, by expanding a variety of industries.

## 4. Conclusions

In this study, the effect of the silicone modifiers of MTMS and TMES on the physical properties of as-prepared flexible silica aerogels (FSAs) was presented. The precursor of the FSAs was synthesized by performing the click reaction between VTMS and EDDET under UV irradiation. Subsequently, the FSAs with/without the modifiers of MTMS and TMES was prepared by performing a conventional sol-gel process (i.e., hydrolysis/condensation reactions). All synthesized products have been characterized by FT-IR and NMR, and their chemical structure was confirmed with ^13^C and ^29^Si solid-state NMR spectroscopy. BET and SEM were used to analyze the pore structure of as-prepared FSAs. Physical properties, such as thermal conductivity, thermal stability, mechanical strength, and surface wettability of FSAs, were determined by the Hot Disk, TGA, durometer/DMA in compression mode, and contact angle measurements, respectively.

It is worth noting, upon the addition of MTMS, the (crosslinking) density of as-prepared FSAs increased. Higher loading of MTMS correlates to a direct increase in the density of the corresponding FSAs. Moreover, upon the addition of MTMS, thermal conductivity, surface hardness, bulk mechanical strength, and surface hydrophobicity of as-prepared FSAs were all increased. On the other hand, upon the addition of 10 wt% of TMES, the thermal conductivity of as-prepared FSAs decreased. However, the surface hydrophobicity and elasticity of the as-prepared FSAs increased.

## Figures and Tables

**Table 1:** Physical properties and porous properties of FSA, FSA-M1, FSA-M5, and FSA-M10.

Sample Code	Feed Composition (wt%)		Thickness (mm)	S_BET_ (m^2^/g) ^b^	V_t_ (cm^3^/g) ^c^	D_p_ (nm) ^d^
	Precursor	Modifier ^a^				
FSA	100	0	6.26 ± 0.1	9.41	0.0312	30.7
FSA-M1	99	1	6.31 ± 0.1	3.21	0.0282	20.4
FSA-M5	95	5	6.30 ± 0.1	1.47	0.0059	15.6
FSA-M10	90	10	6.39 ± 0.1	1.18	0.0026	13.3
FSA-T10	90	10	6.37 ± 0.2	0.23	0.0015	26.7

^a^ MTMS for FSA-M while TMES for FSA-T. ^b^ BET surface area. ^c^ Total pore volume calculated from P/P0 = 0.99. ^d^ Pore diameter.

**Table 2:** Thermal properties, mechanical properties, and surface wettability of FSA, FSA-M1, FSA-M5, and FSA-M10.

Sample Code	Thermal Conductivity (W/mK) ^a^	5 wt% Loss Decomposition Temperature (T_5d_) ^b^	Hardness (N/mm^2^)	Density (g/cm^3^)	Young’s Modulus (MPa) ^c^	Contact Angle (°)
FSA	0.114	86.46	34	0.419	0.35	76.03
FSA-M1	0.120	97.45	35	0.423	0.37	102.49
FSA-M5	0.131	115.98	55	0.448	0.39	113.97
FSA-M10	0.135	121.77	67	0.456	0.52	119.98
FSA-T10	0.091	135.33	20	0.333	0.23	132.35

^a^ As measured by Hot Disk. ^b^ As measured by TGA. ^c^ As measured by DMA.

**Table 3:** Literature status on physical, thermal, mechanical, and hydrophobic properties of silica aerogels.

Sample Code	Density (g/cm^3^)	Thermal Conductivity (W/mK)	Young’s Modulus (MPa)	Contact Angle (°)	Ref.
MTMS-FSA	0.423–0.456	0.120–0.135	0.37–0.52	102.49–119.98	This work
TMES-FSA	0.333	0.091	0.23	132.35	This work
Silsesquioxane aerogel	0.237	0.043	1.31	142.0	[23]
TMCS-silica aerogel	0.225	0.032	12.8	not reported	[40]
polymethylsilsesquioxane aerogels	0.450	0.015	27.0	not reported	[41]
MTMS-silica aerogel with PVMDMS	0.138	0.025	~1.5	136.9	[42]
PVPMS silica aerogel	0.22	0.015	4.7	131.0	[43]

## Data Availability

The data that support the findings of this study are available from the corresponding author, but restrictions apply to the availability of these data, which were used under license for the current study, and so are not publicly available. The data are, however, available from the authors upon reasonable request and with permission of the funding party, Ministry of Science and Technology, Taiwan.
